# Supplementary material for: Tipping points and cascading transitions: methods, principles, and evidence
Source: Natl Sci Rev. 2026 Aug 7;13(20):nwag478. doi: 10.1093/nsr/nwag478 (PMC13715440; doi:10.1093/nsr/nwag478)
Supplement: Supplementary file 1 [file nwag478_supplemental_file.pdf]

Review

National Science Review
XX: main, Year
doi: https://doi.org/10.1093/nsr/XXXX

EARTH SCIENCE

Tipping Points and Cascading Transitions: Methods, Principles, and Evidence

Sheng Fang[1], Ziyan Wang[1], Jürgen Kurths[2,3,*] and Jingfang Fan[1,2,*]

ABSTRACT

This review synthesizes recent advancements in understanding tipping points and cascading transitions within the Earth system, framing them through the lens of nonlinear dynamics and complexity science. It outlines the fundamental concepts of tipping elements, large-scale subsystems like the Atlantic Meridional Overturning Circulation and the Amazon rainforest, and classifies tipping mechanisms into bifurcation-, noise-, and rate-induced types. The article critically evaluates methods for detecting early-warning signals, particularly those based on critical slowing down, while also acknowledging their limitations and the promise of non-conventional indicators. Furthermore, we explore the significant risk of cascading transitions between interacting tipping elements, often modeled using conceptual network models. This shows that such interactions can substantially increase systemic risk under global warming. The review concludes by outlining key challenges related to data limitations and methodological robustness, and emphasizes the promising role of artificial intelligence and complex network science in advancing prediction and risk assessment of Earth system tipping dynamics.

Keywords: Tipping point, Tipping cascade, Bifurcation, Early-warning signals, Complex network

[1]School of Systems Science, Beijing Normal University, 100875 Beijing, China; [2]Potsdam Institute for Climate Impact Research, 14412 Potsdam, Germany; [3]Department of Physics, Humboldt University Berlin, 10099 Berlin, Germany

*Corresponding authors. Email: kurths@pik-potsdam.de, jingfang@bnu.edu.cn.

Received: XX XX Year; **Revised:** XX XX Year; **Accepted:** XX XX Year

Introduction

The ocean, covering about 71% of Earth's surface, is a cornerstone of the Earth system, underpinning global climate regulation, sustaining marine biodiversity, and supporting socioeconomic development. It has absorbed nearly 93% of the excess heat and about 30% of anthropogenic CO_2 emissions [1,2], harbors approximately 2.2 million species [3], and contributes around USD 2.6 trillion annually to the global economy [4]. However, under the combined pressures of global warming and human activities, the ocean is experiencing profound and systemic changes, including sea-level rise, warming, acidification, and deoxygenation [5]. These interconnected processes are eroding marine ecosystem resilience and may push subsystems toward irreversible regime shifts. This threat is especially acute in coastal regions owing to their position at the land–ocean boundary and heightened sensitivity to environmental changes. Since they are exposed simultaneously to terrestrial, marine, and human-induced disturbances, coastal systems often face amplified cumulative impacts that can accelerate ecological degradation and push them closer to critical thresholds. For example, global warming and anthropogenic nutrient inputs have caused severe deoxygenation in the Baltic Sea [6], which reduces fishery yields and promotes harmful algal blooms, which are extremely difficult to reverse through governance or intervention.

The marine environment exemplifies a complex adaptive system, in which physical, biological, and socioeconomic components are dynamically coupled through nonlinear feedbacks operating across multiple spatial and temporal scales. Understanding such intertwined processes requires a holistic framework, as provided by Earth System Science (ESS). ESS conceptualizes the planet as an integrated system of interacting physical, chemical, biological, and anthropogenic subsystems [7]. It seeks to reveal how natural processes and human activities jointly shape global dynamics, encompassing the climate system, biogeochemical cycles, and ecosystems. By integrating insights from geology, meteorology, oceanography, ecology, and the social sciences, ESS provides a com-

© The Author(s) Year. Published by Oxford University Press on behalf of China Science Publishing & Media Ltd. This is an Open Access article distributed under the terms of the Creative Commons Attribution License (http://creativecommons.org/licenses/by/4.0/), which permits unrestricted reuse, distribution, and reproduction in any medium, provided the original work is properly cited.

prehensive framework for addressing grand challenges in sustainability, resilience, and planetary boundaries, and for guiding the informed management of Earth's complex systems.

Within the ESS framework, the concept of *tipping points* has attracted increasing attention [8,9]. A tipping point refers to a critical threshold beyond which small perturbations can trigger large and often irreversible changes in a system's state. The corresponding large-scale subsystems of the Earth, which are known as *tipping elements*, include the Atlantic Meridional Overturning Circulation (AMOC), the Amazon rainforest, the Greenland Ice Sheet (GIS), and the West Antarctic Ice Sheet (WAIS), etc. Various methods, including early-warning signals (EWS), have been designed to anticipate the tipping points [10]. Importantly, these elements are not independent; their interactions can induce cascading effects that amplify systemic risk [11]. For instance, rapid Arctic sea-ice loss introduces freshwater into the North Atlantic, weakening the AMOC. This slowdown can reduce rainfall over South America, accelerating the Amazon forest dieback. The resulting carbon release enhances global warming, potentially destabilizing the West Antarctic Ice Sheet [9,12]. Recent assessments suggest that several major tipping elements are approaching, or may already have crossed, their critical thresholds [9], and that exceeding 1.5 °C of global warming could trigger multiple, interconnected tipping events [13]. Recent evidence indicates that warm-water coral reefs are already confronting unprecedented mortality, suggesting that these ecosystems may have crossed critical thermal thresholds [14].

In this review, we provide an integrative synthesis that connects diverse tipping mechanisms within a comprehensive framework. We systematically bridge the theoretical foundations of tipping dynamics with state-of-the-art detection methodologies, explicitly expanding beyond classical temporal and spatial EWS to encompass advanced multivariate indicators. Crucially, we extend this framework to interconnected systems by highlighting recent, timely developments in cascading transitions, including overshoot-induced cascade risks, indirect tipping pathways, and non-local interactions. Finally, we critically examine emerging frontiers, emphasizing how explainable and physics-informed artificial intelligence, together with complex network science, can overcome traditional methodological limitations. Ultimately, our contribution lies in weaving these rapidly evolving strands of research into a cohesive paradigm, thereby advancing the prediction, understanding, and risk assessment of systemic transitions in the interconnected Earth system.

Tipping point and tipping element

The concept of "tipping point" originated from observations of abrupt sociological changes, capturing how small perturbations can lead to disproportionately large outcomes [15]. Over time, the term gained traction beyond the social sciences and has been increasingly adopted across diverse disciplines [10], including ESS. A prominent application arises in climate science, where such thresholds are closely linked to what was previously termed *abrupt climate change* [16].

Since the 1970s, the understanding of abrupt climate change has evolved from a theoretical notion to an empirically grounded paradigm. This shift was largely driven by high-resolution paleoclimatic archives, which revealed that Quaternary climate history was punctuated by rapid, high-magnitude oscillations, such as the Younger Dryas stadial [17]. Broecker [18] played a pioneering role in advancing the hypothesis that the climate system exhibits nonlinear behavior capable of rapid reorganization, particularly through perturbations to the AMOC. Subsequent syntheses of ice-core and marine sediment records further reinforced this view, demonstrating the recurrent nature of abrupt transitions throughout Earth's history [16].

Building upon this foundation, Lenton *et al.* [8] formally defined a tipping point as the critical threshold of a control parameter, ρ_c, beyond which an infinitesimal perturbation induces a significant change, $\hat{F}$, in the system state F within a finite observation time T. This definition can be expressed as

$$|F(\rho \geq \rho_c + \delta\rho|T) - F(\rho_c|T)| \geq \hat{F} > 0. \quad (1)$$

They further introduced the concept of *climate tipping elements*, referring to subcontinental-scale components of the Earth system that may cross a tipping point and transition into a qualitatively new state under the influence of climate change or anthropogenic forcing. These tipping elements are of particular policy relevance, as their activation could amplify global temperature rise and destabilize other parts of the system. Moreover, these elements are not independent, where transitions in one tipping element can trigger cascading effects across others, potentially leading to widespread and irreversible transformations of the Earth system.

A typical example of a tipping element is the Amazon rainforest, which harbors over 10% of Earth's terrestrial biodiversity and serves as one of the planet's largest carbon sinks, absorbing vast amounts of atmospheric carbon dioxide through photosynthesis [19]. However, under the combined pressures of climate change and intensifying human activities, extensive regions of the Amazon rainforest are projected to undergo large-scale degradation and dieback in the coming decades [20]. A recent study [21] suggests that by 2050, approximately 10–47% of the Amazon rainforest could be exposed to compounding disturbances, potentially triggering abrupt ecosystem transitions and amplifying regional climate change. Notably, the seminal paper in Ref. [8] identified the Amazon rainforest as a potential tipping element of the Earth system, and its collapse could drive an additional global temperature rise of 3–4 °C, which underscores its pivotal role in regulating planetary stability.

Faced with such grave risks from negative tipping elements, a promising avenue for climate mitigation lies in harnessing *positive tipping points* (PTPs), which deliberate socio-technical shifts where targeted interventions initiate self-reinforcing feedbacks that accelerate decarbonization [26]. Unlike the often irreversible Earth system tipping points, PTPs arise when reinforcing mechanisms, such as economies of scale, learning-by-doing, and social diffusion, overcome the inertia of incumbent high-carbon systems. Empirical examples are emerging: the rapid adoption of electric vehicles in several regions exhibits EWS of an approaching technological transition [27]. Moreover, strategic policy interventions, such as mandates and phase-out regulations, can create "super-leverage points", triggering cascading changes across coupled sectors like transport and power [28]. Thus, alongside managing risks from climatic tipping elements, proactive efforts to identify and trigger PTPs offer a crucial pathway toward stabilizing the Earth system.

Bifurcation and tipping types

To model the tipping process, we consider a generalized dynamical system,

$$\frac{dx}{dt} = F(x, \rho(t)), \tag{2}$$

where x represents the system state variable and $\rho(t)$ is a time-dependent control parameter. In the special case where ρ is constant, Eq. (2) reduces to an autonomous system that may evolve toward a quasi-static attractor. Based on the underlying mechanisms, whether state transitions are triggered by internal dynamics or external perturbations, tipping phenomena can generally be classified into three causally distinct types: (i). *bifurcation-induced tipping* (B-tipping), (ii). *noise-induced tipping* (N-tipping), and (iii). *rate-dependent tipping* (R-tipping) [14, 22].

(i). B-tipping occurs when a slow, continuous variation in system parameters (e.g., external forcing or climate trends) drives the system through a bifurcation, causing a stable state, such as a fixed point or a periodic orbit, to lose stability or vanish. This loss of stability leads to an abrupt transition to an alternative attractor. (ii). In contrast, N-tipping arises in multistable systems, where stochastic fluctuations (e.g., interannual variability in the climate system) can push the system across a potential barrier, inducing a noise-driven transition from one stable state to another. (iii). R-tipping occurs when the rate of parameter change is sufficiently rapid that the system cannot adiabatically follow its quasi-static equilibrium. As a result, the system departs from the expected attractor before the bifurcation point is reached, or it may delay the transition, which leads to an early or late tipping event.

To illustrate these concepts, we employ the "zero-dimensional" global energy balance model [23,24], expressed as

$$\begin{aligned} dT &= f(T)\,dt + \sqrt{\nu}\,dW, \\ f(T) &= c_0[-T^4 + c_1\mu(b_2T^2 - a_2)]. \end{aligned} \tag{3}$$

This model describes the temporal evolution of the global mean surface temperature T, representing the balance between incoming solar radiation and outgoing longwave radiation. The term dW denotes a normalized Wiener process (white noise) with amplitude $\sqrt{\nu}$, capturing stochastic perturbations from the environment. All parameters are scaled such that $f(T)$ has dimensions of $\mathrm{K\,yr^{-1}}$. Under these assumptions, the equilibrium-state parameters can be computed as $c_0 = 1.10869 \times 10^{-8}\mathrm{K}^{-3}\,\mathrm{yr}^{-1}$ and $c_1 = 9.7137 \times 10^9\mathrm{K}^4$.

The parameters μ, b_2, and a_2 are treated as control variables. Table 1 summarizes the parameter settings used to simulate different types of tipping, while the corresponding time evolutions are shown in Fig. 1. For the B-tipping case, the system evolves toward a stable fixed point when far from the critical threshold. As the tipping point is approached, the distance between the stable and unstable equilibria de-

Parameter	B-tipping	N-tipping	R-tipping
μ	$\frac{d\mu}{dt} = -4 \times 10^{-4}$, $\mu(0) = 1.0$	1.0	1.0
$b_2 (\times 10^{-5}\text{K}^{-2})$	1.04	1.690	$(1-\lambda_1)b_{\text{ini}} + \lambda_1 b_{\text{fin}}$
a_2	0.2	0.6927	$(1-\tilde{\rho}_1(\lambda_1))a_{\text{ini}} + \tilde{\rho}_1(\lambda_1)a_{\text{fin}}$
ν	0	1.0	0

Table 1. Parameter values for various type of tipping via Eq. (3), which originates from Ref. [22]. For B-tipping, the noise is absent with $\nu = 0$ and the parameter μ is decreased from 1 at a rate of 4×10^{-4} per year. For N-tipping, the parameter is fixed to 1.0 and the amplitude $\nu = 1$. For R-tipping, the initial and final values of parameters b_2 are fixed as $b_{\text{ini}} = 1.69 \times 10^{-5}$ and $b_{\text{fin}} = 1.835 \times 10^{-5}$. The parameter λ controls the ariability of b_2, which is the solution of equation $d\lambda_1/dt = \rho_1\lambda_1(1-\lambda_1)$. In the simulation, the initial parameter of λ_1 is set to 10^{-6}. A similar case is applied for a_2 with $a_{\text{ini}} = 0.6927$ and $a_{\text{fin}} = 0.8168$, and $\tilde{\rho}_1(\lambda_1)$ is the controlling parameter such that $(c_1\mu b_2)^2 - 4c_1\mu a_2$ is constant at a rate proportional to ρ_1.

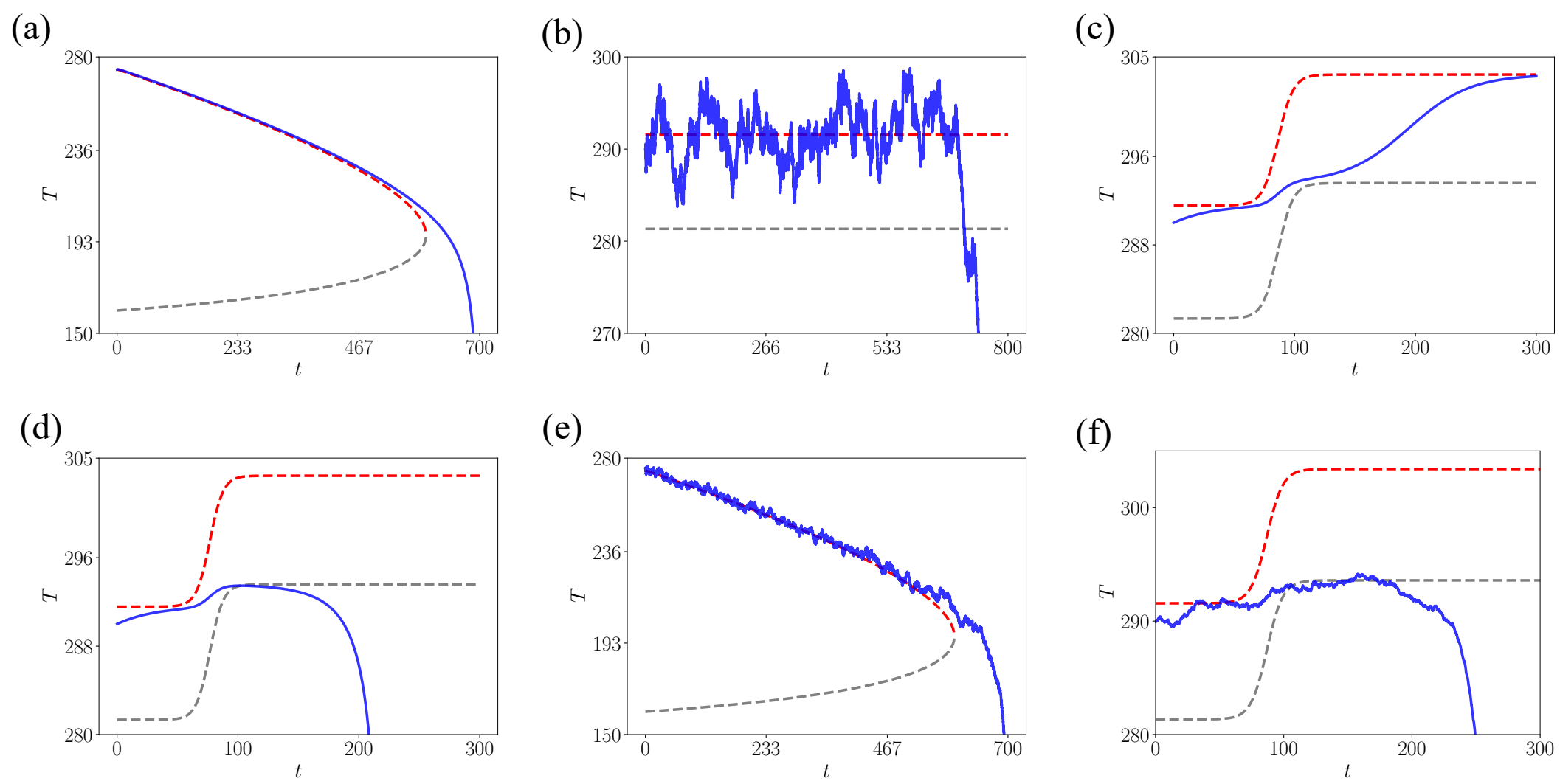

Figure 1. Illustrations of trajectories for the Sutera-Fraedrich model Eq. (3) [23,24] showing the presence of various types of tipping with (a) B-tipping, (b) N-tipping, (c)-(d) R-tipping, (e) B-tipping (with noise), and (f) R-tipping (with noise). Panels (a)-(d) are replotted from Figure 7 in Ref. [22], and the controlled parameters are summarized in Table 1. The horizontal axis is time with dimension yr, and the vertical axis is the temperature with dimension K. The solid lines show system trajectories while the dashed lines show the location of the fixed point, where red dashed denotes the stable fixed point and the gray dashed line is for the unstable fixed point. For R-tipping, the parameter ρ takes 0.16 for (c) and 0.18 for (d), and the critical rate is approximately 0.175. Panels (e) and (f) show two different types of combined tipping types. Panel (e) illustrates noisy B-tipping, using the same parameters as in Panel (a) but with a noise intensity of $\sqrt{\nu} = 1.0$. Panel (f) displays noisy R-tipping, with parameters identical to those in Panel (c) and a noise intensity of $\sqrt{\nu} = 0.14$. The codes for simulations and plotting are available at [25].

creases, causing the system trajectory to deviate from the stable branch. In the N-tipping scenario, the system exhibits bistability in the absence of noise. Before the transition occurs, the state fluctuates around the stable fixed point but may stochastically jump to the alternative attractor (e.g., near $T \approx -300$ K) once noise is introduced. R-tipping differs fundamentally from the previous two types: the system initially follows a quasi-steady trajectory between the stable and unstable branches [22]. When the rate of parameter variation $\rho(t)$ exceeds a critical threshold, the system departs from the stable branch before reaching the bifurcation point, triggering a rapid transition, as illustrated in Fig. 1(c).

Furthermore, different types of tipping points can interact and combine in ways analogous to real-world scenarios, with their behaviors mutually influencing one another. In Figs. 1(e)-(f), we illustrate B-tipping and R-tipping under the influence of noise. For B-tipping (Fig. 1(e)), the system fluctuates near a stable fixed point, and the transition threshold remains largely unchanged, demonstrating strong robustness against stochastic perturbations. In contrast, for R-tipping, a comparison between Figs. 1(c) and (f), which share identical parameters except for the addition of noise with amplitude $\sqrt{\nu} = 0.14$ in panel (f), reveals that the system becomes notably more fragile. This increased fragility occurs even under a relatively weak noise level ($\sqrt{\nu} = 0.14$), which is substantially smaller than the amplitude ($\sqrt{\nu} = 1.0$) applied in the B-tipping case. Analyzing such mixed tip-

ping events, where multiple mechanisms interplay, presents stronger theoretical and diagnostic challenges.

Beyond these basic types, more refined frameworks have been proposed to classify tipping transitions, especially when multiple timescales or spatial structures are at play. For instance, Ref. [29] categorizes transitions into three types, the state transition, basin transition, and distribution transition, which are based on the interplay among the timescale of parameter variation (τ_λ), the timescale of state transition (τ_{tran}), the ergodic timescale (τ_{ergo}), and the noise intensity. Moreover, when spatial heterogeneity and subsystem couplings are explicitly considered, such as in coastal regions, Ref. [30] identified two additional tipping types: space-driven tipping and information-driven tipping, highlighting the roles of explicit spatial structure and cross-component interactions in mediating regime shifts.

Early-warning signals and tipping point detection

Since tipping events can cause profound and often irreversible shifts in system states, detecting and anticipating their onset is of paramount importance. Yet, the intrinsic complexity of empirical systems often makes it difficult to determine the critical threshold in advance. Fortunately, many complex systems exhibit *universal* signatures, such as rising variance, autocorrelation, and skewness, as they approach critical transitions. These phenomena are collectively referred to as EWS [10]. A range of EWS detection methods have been developed and are summarized in Table 2, following Ref. [31]. Among these approaches, one of the most widely applied is based on the concept of *critical slowing down* (CSD) [32], which describes the progressive decline in a system's recovery rate as it nears a bifurcation point in B-tipping scenarios. Indicators derived from CSD, such as variance, temporal and spatial correlations, and recovery rate, are particularly useful for detecting imminent transitions, as summarized in Table 2.

To illustrate the underlying mechanism, consider a stochastic saddle-node bifurcation system [33,34] governed by

$$\frac{dx}{dt} = ax^3 + bx + \phi(\rho) + \xi(t), \tag{4}$$

where $\phi(\rho)$ represents a control parameter, and $\xi(t)$ denotes Gaussian white noise. This nonlinear dynamic model can be used to describe the evolution of tipping elements, under the assumption that the interaction within the tipping element can be regarded as stochastic noise, which is reasonable since the scale of climate tipping elements is so large, with subcontinental scale. In the absence of noise, stability analysis yields the critical thresholds $\phi_c^{\pm} = \pm\sqrt{-4a^3b^3/27a^4}$, under the intrinsic condition $a < 0$ and $b > 0$. When $\phi < \phi_c^-$ or $\phi > \phi_c^+$, the system possesses a single equilibrium; for $\phi_c^- < \phi < \phi_c^+$, two stable equilibria coexist with one unstable state. As ϕ approaches ϕ_c^+, all equilibria merge and vanish, marking a classical saddle-node bifurcation.

Let us consider a specific case with $a = -1$ and $b = 1$, for which $\phi_c = \pm\frac{2\sqrt{3}}{9}$. Near ϕ_c^-, the equilibrium position is $x_c = \frac{\sqrt{3}}{3}$. Introducing a small perturbation ϵ around equilibrium, $x = x_c + \epsilon$, the local linearization gives

$$\frac{dx_c}{dt} + \frac{d\epsilon}{dt} = f(x, \phi), \tag{5}$$

$$f(x, \phi) \approx f(x_c, \phi) + \left.\frac{\partial f(x, \phi)}{\partial x}\right|_{x_c} \epsilon + O(\epsilon^2). \tag{6}$$

For $x \to x_c$, the recovery rate is $\lambda(\phi) = \left.\frac{\partial f(x,\phi)}{\partial x}\right|_{x_c} = -3x_c^2 + 1$. At the critical point, $\lambda(\phi) \to 0$, implying that small perturbations decay increasingly slowly, a hallmark of CSD.

This CSD manifests as increasing temporal and spatial correlations. For a discrete time series, the lag-1 autocorrelation (AC1) [10,34] is defined as

$$\text{AC1} = \frac{\sum_{t=2}^{T}(x_t - \bar{x})(x_{t-1} - \bar{x})}{\sum_{t=1}^{T}(x_t - \bar{x})^2}. \tag{7}$$

Assuming small perturbations ($\bar{x} \approx x_c$), we have $\text{AC1} \approx e^{\lambda}$ via Eqs. (5) (6). As the system approaches the threshold, $\lambda \to 0$ and hence $\text{AC1} \to 1$. Similarly, for perturbations obeying

$$x_{t+1} - \bar{x} = e^{\lambda}(x_t - \bar{x}) + \sigma\xi(t), \tag{8}$$

where σ denotes the amplitude of the white noise, the variance satisfies

$$\text{Var}(x_{t+1} - \bar{x}) = e^{\lambda}\text{Var}(x_t - \bar{x}) + \sigma. \tag{9}$$

Thus, $\text{Var} = \sigma/(1 - e^{\lambda})$, which diverges as $\lambda \to 0$, signaling proximity to the critical point.

Another commonly used technique is *detrended fluctuation analysis* (DFA) [35], designed to quantify long-term correlations and scaling properties in noisy, nonstationary time series. DFA removes local trends to estimate a scaling exponent α, which approaches unity

near a tipping point. Unlike AC1, which captures short-term (lag-1) correlations, DFA reveals long-range memory effects and helps mitigate false positives arising from slow drifts or nonstationarity [36,37]. However, it generally requires longer time series and is computationally more demanding.

In Fig. 2, we illustrate the dynamics of Eq. (4) and its corresponding CSD indicators. The control parameter is set as $\phi = -1 + 0.004t$ [38]. As the system approaches the bifurcation, both AC1 and variance increase markedly, with AC1 reaching a plateau and subsequently declining after the transition has occurred. Further, the abrupt change near the tipping point of the original data causes the plateau of the EWS indicators, which could be designed to detect the tipping point.

EWS approaches have been widely implemented in the analysis of diverse climate subsystems [11,34,39]. For instance, using a century-long dataset (1900–2020) of sea surface temperature and salinity from the subpolar North Atlantic, the author applied statistical EWS indicators analysis and presented empirical evidence suggesting that the AMOC may be approaching a tipping point [34]. Their analysis revealed a pronounced increase in both autocorrelation and variance over time, hallmarks of CSD that indicate a loss of dynamical resilience. These results suggest that the AMOC has been progressively weakening, particularly since the mid-20th century, and may be nearing a tipping point. While the precise timing of a potential collapse remains uncertain, this study provides the first robust empirical warning that such an event could be imminent, underscoring the urgent need for enhanced observation and proactive climate mitigation efforts.

In addition to temporal indicators, CSD leaves distinct signatures within the spatial patterns of connected subsystems approaching a critical threshold [40,41]. When a spatially extended system nears a tipping point, its localized recovery rate from external perturbations diminishes, causing neighboring regions to become increasingly synchronized. This spatial manifestation of CSD leads to a marked increase in spatial autocorrelation, meaning that adjacent patches exhibit highly similar states [40]. Furthermore, the slowed recovery amplifies spatial fluctuations, resulting in elevated spatial variance and prominent changes in spatial skewness [41]. Other spatial metrics, such as an expanding spatial recovery length, have also been identified as robust leading indicators of impending regime shifts [42]. Crucially, these spatial EWS offer a significant advantage over their temporal counterparts: they can often be derived from a single or a few high-resolution spatial snapshots, such as those obtained via satellite remote sensing, thereby circumventing the strict requirement for long, uninterrupted historical time series [43].

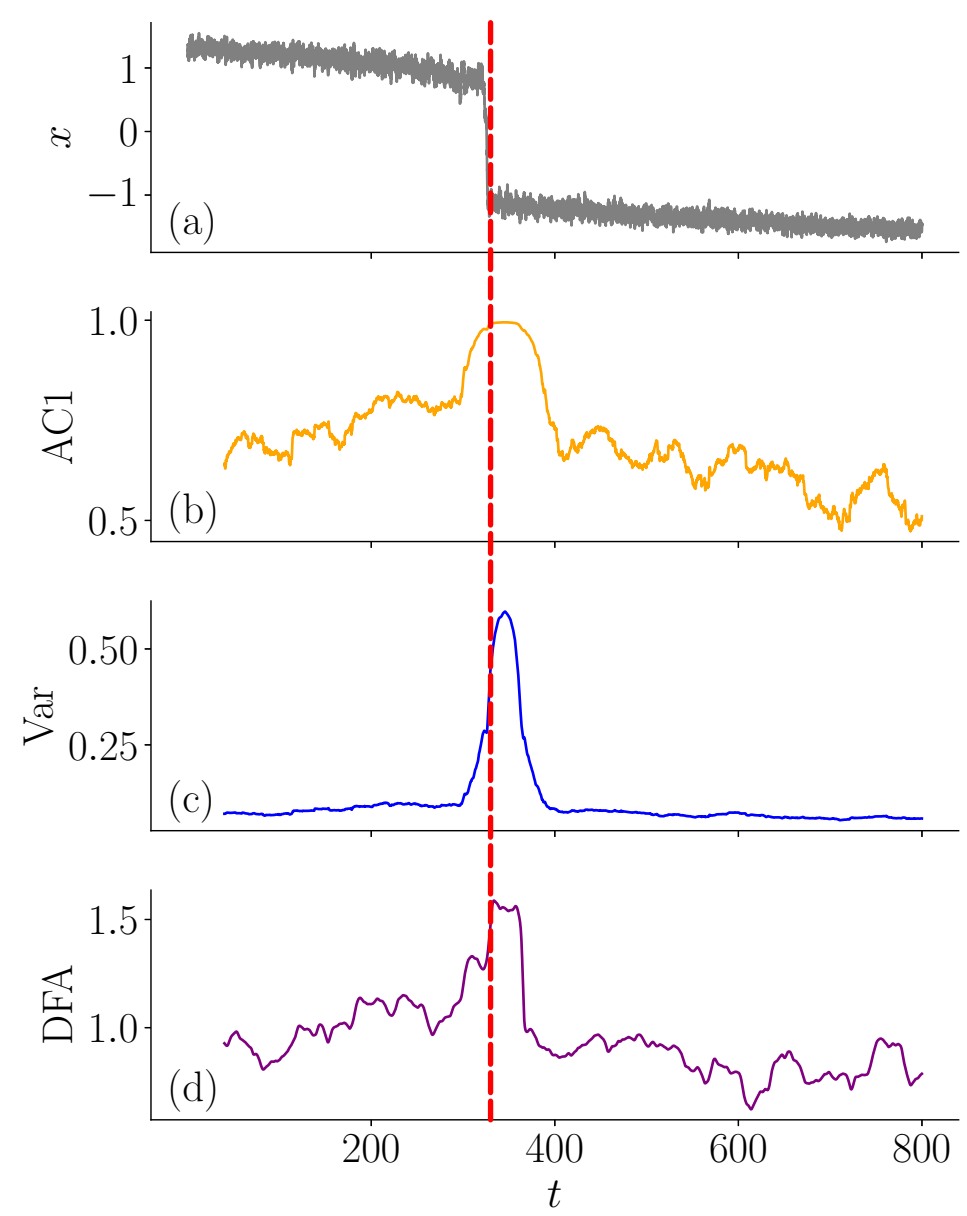

Figure 2. Illustration of the EWS of the tipping dynamic. The B-tipping dynamics is governed by Eq. (4) (a) with three types of EWS indicators (b) AC1, (b) Var, and (d) DFA. For each EWS indicator, we set the window width to 400. The vertical dashed line corresponds to the location of the tipping point.

Moreover, these indicators are particularly valuable in large-scale climate and ecological systems characterized by strong spatial organization. Compared to purely temporal approaches, incorporating spatial information enhances predictive capability and improves the robustness of EWS detection, especially when observational records are relatively short or heavily obscured by noise [40]. Building on these spatial methodologies, recent advancements have expanded the application of CSD theory to highly complex spatio-temporal domains, including interconnected ecosystems and transient landforms [44]. For instance, a novel framework based on eigenvalue tracking and Floquet multipliers has been developed to quantify resilience directly from spatio-temporal data without the prerequisite of removing underlying trends or seasonality. This innovative approach not only successfully recovers spatially explicit destabilization patterns in the Amazon rainforest and predicts glacier surge onsets, but also demonstrates remarkable robustness to the varying noise levels inherently asso-

ciated with multi-sensor satellite records.

Even though CSD-based EWS have been successfully applied across a wide range of disciplines, they still face notable limitations. These methods are fundamentally grounded in the assumption that perturbations can be linearized near an equilibrium state, an assumption that holds primarily for B-tipping scenarios. However, this premise breaks down in the cases of N-tipping and R-tipping. In N-tipping, stochastic fluctuations exert strong, nonlinear influences that cannot be captured by linearization. In R-tipping, the system trajectory departs from any quasi-static equilibrium due to the rapid variation of external parameters, rendering linearization-based approaches invalid.

To address these shortcomings, several non-CSD-based EWS indicators have been proposed, which do not rely on the approach of the recovery rate toward zero. Instead, they detect other high-order statistical signatures, which emerge as the system nears a tipping point and are particularly suitable for systems exhibiting N-tipping or evolving far from quasi-static equilibrium. For example, skewness [45], which quantifies the asymmetry of the system-state distribution, can reveal nonlinear shifts toward a tipping point by identifying increasing occurrences of extreme deviations in one direction. Another notable indicator is flickering [46], a phenomenon in which the system intermittently switches between alternative stable states under stochastic forcing. Such behavior reflects the progressive loss of resilience as the system begins to explore adjacent basins of attraction. Together, these non-CSD-based indicators provide valuable diagnostic tools for anticipating critical transitions in systems where traditional CSD-based methods fail to apply.

Another prominent information-theoretic, non-CSD-based indicator is the Fisher information, which quantifies the dynamic order and certainty of a system's state distribution without relying on CSD [47,48]. In complex multivariate systems, a stable regime is characterized by high dynamic order and bounded variance, yielding a high and steady value of Fisher information [49]. However, as the system approaches a critical threshold and its resilience deteriorates, the state trajectories become increasingly erratic and disordered [50]. This progressive loss of system organization is reflected as a steady decline in Fisher information, providing a robust EWS of an impending regime shift well before the actual transition occurs [48]. Furthermore, Fisher information is highly advantageous because it can effectively collapse complex, multivariate time-series data into a single systemic index, offering a reliable sentinel for both temporal and spatial transitions in ecological and socio-economic networks [51]. The commonly used EWS indicators are summarized in Table 2.

Cascading transitions

From a risk-assessment perspective, the catastrophic impact of crossing a tipping point arises not only from the transition of an individual subsystem, but also from its capacity to trigger cascading effects across other tipping elements through complex interactions. Such cascades can propagate through interconnected components of the Earth system, including the cryosphere, biosphere, and ocean circulation, forming feedback loops that amplify the initial perturbation and may lead to large-scale, potentially irreversible consequences. A recent study indicates that exceeding a global warming threshold of 1.5°C could trigger multiple climate tipping points [13], and that some critical thresholds might even lie below this level [8]. This highlights that the cumulative risk of interacting tipping elements is substantially greater than that inferred from analyzing them in isolation, especially when considering synergistic effects and domino-like cascades of tipping events.

Although the terms "cascade" and "domino effect" are often used interchangeably across disciplines, a key distinction lies in whether causality is a necessary condition for a cascade [33,76]. Klose *et al.* [77] proposed three distinct patterns of multiple tipping dynamics: the *domino cascade*, the *two-phase cascade*, and the *joint cascade*. To illustrate these, we consider a leading system X_1 and a following system X_2, driven by external control parameters r_1 and r_2, respectively. Typically, the driver r_2 for the following system X_2 is set to be a constant.

As r_1 changes, the leading system X_1 may tip and, through direct coupling, induce tipping in X_2, a process known as a *domino cascade*. This form emphasizes causality, where tipping signals propagate from the leading to the following system through explicit interaction, and has been widely used to model signal transmission between tipping elements in the Earth system [12,78]. In contrast, a *two-phase cascade* describes a situation in which both systems X_1 and X_2 cross their tipping points sequentially as r_1 varies, yet the tipping of X_1 does not immediately cause that of X_2; rather, further sustained change in r_1 leads to the subsequent tipping of X_2. Thus, causality is not a necessary condition

Table 2. Summary of methods to detect EWS for tipping points. The table is adapted from Ref. [31].

**CSD-based**	Variance (temporal/spatial) [32,34]<br>Autocorrelation (temporal/spatial) [32,34]<br>Return rate and/or time (temporal) [34]<br>Detrended fluctuation analysis (temporal) [35,52]<br>Spectral reddening (temporal) [53]<br>Variance–covariance eigenvalue (temporal) [54]<br>Dynamic eigenvalue (temporal) [55]<br>repair time (spatial) [10,56]<br>Empirical orthogonal function based variance (spatial/temporal) [57]<br>Generalized models (temporal) [58]<br>Time-varying AR(p) models (temporal) [59]<br>Probabilistic time-varying AR(p) (temporal) [60]<br>Detrended cross-correlation analysis (temporal) [33,61]
**Non-CSD-based**	Skewness (temporal/spatial) [41,45]<br>Kurtosis (temporal) [45]<br>flickering (temporal) [46]<br>Fisher information (temporal) [47,48]<br>Potential analysis (temporal) [62]<br>Conditional heteroscedasticity (temporal/spatial) [63]<br>Energy landscape-flux theory (temporal/spatial) [64,65]<br>Turing patterns (spatial) [43]<br>Patch size distributions (spatial) [66]<br>Hurst exponent (spatial/temporal) [67]<br>Trait statistical changes (temporal) [68]<br>Threshold AR(p) models (temporal) [69]<br>Machine learning approach (temporal) [70,71]<br>Dynamical network biomarker (temporal/spatial) [72]<br>Climate networks (temporal/spatial) [73]<br>Koopman/Kolmogorov operator (temporal) [74]<br>Dynamic mode decomposition (temporal/spatial) [75]

for this type of cascade. This mechanism has been invoked to explain the consecutive tipping of the AMOC (as X_1) and the Antarctic Ice Sheet (as X_2) during the Eocene–Oligocene transition, accounting for the stepwise shifts observed in oxygen isotope records [33,79]. In a *joint cascade*, two systems X_1 and X_2 tip simultaneously, as observed in some spatially extended bistable ecosystems [40,80].

This distinction is useful because only the first class encodes a direct propagation by construction, while the others can arise from common forcing, timescale separation, or mutual destabilization. Recent work has broadened this taxonomy further. Indirect tipping refers to transitions mediated by intermediate state changes or environmental pathways rather than by a single direct trigger; indirect cascade tipping extends this logic to multi-step pathways; and cascade hopping highlights cases in which the perturbation effectively bypasses an apparently intermediate subsystem and activates a more distant one [81,82]. Closely related work on accelerating cascades emphasizes that one tipping event can shorten the timescale to subsequent ones, even when the network pathway is not strictly local [83]. These concepts are especially relevant for Earth-system applications, where teleconnections, unequal response times, and mediated feedbacks can make cascade pathways nonlocal and non-intuitive.

Compared with single-element tipping processes, compound tipping involving cascade effects poses far greater challenges for scientific analysis. These challenges span the identification, diagnosis, early warning, and prediction of tipping events, as well as the mathematical modeling and quantitative characterization of cascade dynamics themselves. In Ref. [33], the authors developed a dynamical-systems framework to describe deterministic cascading processes between coupled subsystems. The conceptual model can be formulated as

$$\begin{cases} \dfrac{dx}{dt} = F_{X_1}(x, \rho), \\ \dfrac{dy}{dt} = F_{X_2}(y, x), \end{cases} \tag{10}$$

where x and y represent the state variables of the

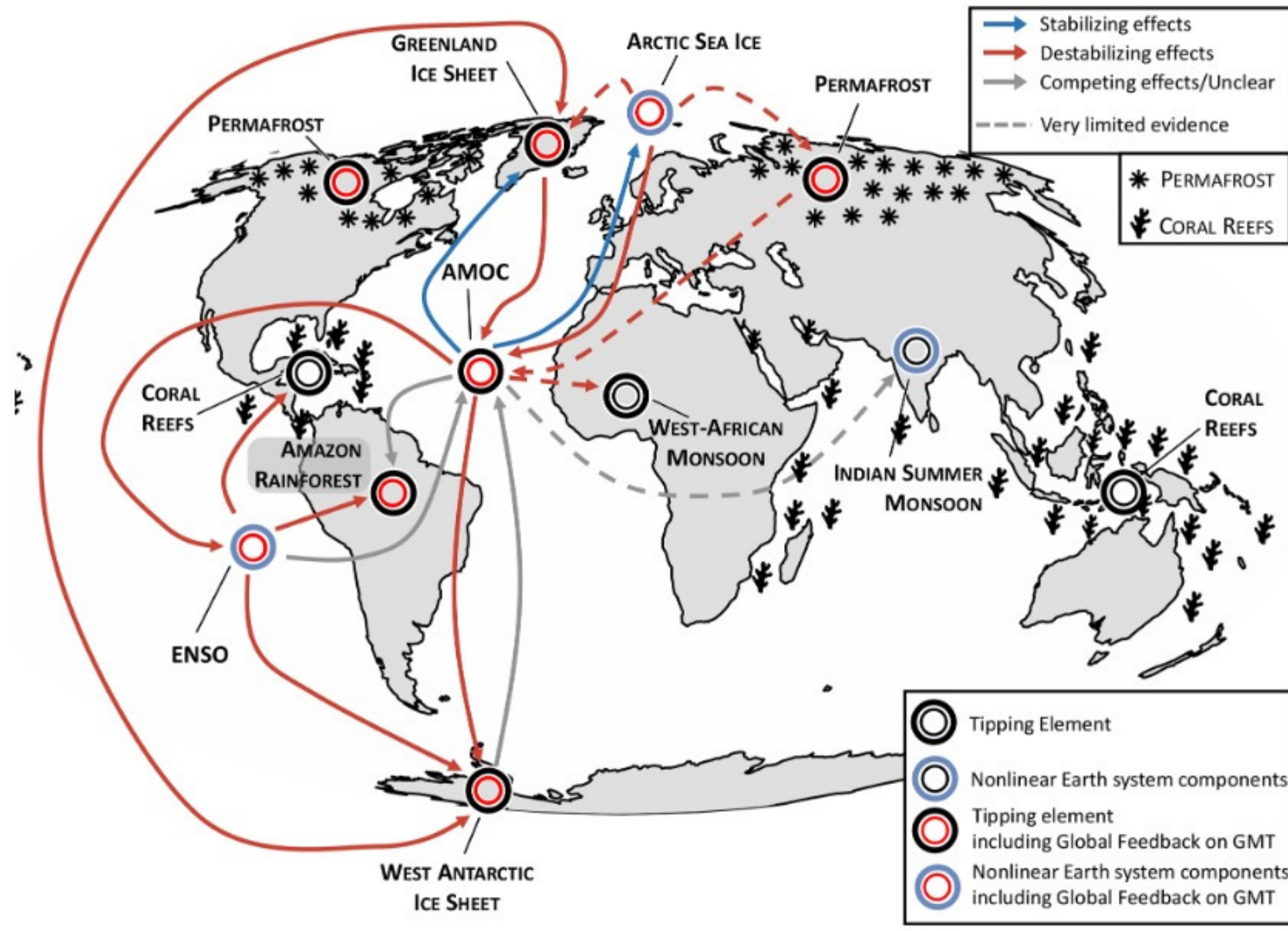

Figure 3. Illustration of interactions between tipping elements on a world map, and this figure originates from Ref. [84]. All tipping elements discussed in this review article are shown together with their potential connections. The causal interactions links can have stabilizing (blue), destabilizing (red), or unclear (gray) effects. Tipping elements that exert a notable feedback on global mean temperature when they tip are denoted by a red inner ring. This temperature feedback can be positive (i.e., amplifying warming, as likely for the permafrost, the Arctic sea ice, the GIS and WAIS, the Amazon rainforest, and ENSO) or negative (i.e., dampening warming, as likely for the AMOC).

leading system X_1 and the following system X_2, respectively. The functions F_{X_1} and F_{X_2} characterize the underlying bifurcation types, such as fold or Hopf bifurcations. The leading system X_1 is driven by an external control parameter ρ, while the following system X_2 is influenced by the state of X_1 through linear coupling. Here ρ denotes the external control parameter that organizes the bifurcation structure; in autonomous analyses it is typically fixed within a given simulation and varied only across parameter sweeps or quasi-static experiments. Numerical simulations demonstrated that conventional EWS may become unreliable for the following subsystem. Specifically, signals transferred from X_1 to X_2 can induce apparent increases in autocorrelation and variance in X_2, even when the latter remains far from its own critical threshold. Consequently, such changes may produce false indications of destabilization and obscure the distinction between internally generated and externally induced transitions. For interacting subsystems, univariate indicators may therefore fail to capture changes arising from coupling structures and collective dynamics. Multivariate and interaction-aware EWS methods provide an important extension in this context. For example, detrended cross-correlation analysis (DCCA) quantifies scale-dependent correlations between nonstationary time series [61], enabling the detection of increasing co-fluctuations among subsystems prior to transitions. Similarly, multivariate covariance-based indicators and network-based approaches can characterize changes in collective resilience, synchronization, and teleconnection structures across interacting components [31,85]. These methods are particularly relevant for identifying cascading behavior in interconnected systems. However, their performance remains sensitive to factors such as detrending procedures, window-length selection, common external drivers, and pronounced nonstationarity.

Although the current framework is primarily designed for interactions between two coupled systems, it can be readily generalized to multiple systems [81,83]. For instance, the following system X_2 can itself be treated as the leading system for a subsequent subsystem X_3. By iterating this structure, one can model cascades across multiple tipping elements, yielding clearly defined and directed cascade pathways. However, this framework overlooks two important aspects: (i). first, the direct influence of external drivers (such

as global temperature) on the following systems, and (ii). second, the representation of feedback loops among various tipping elements due to the tipping cascade. Both aspects are critical for realistically modeling interactions among tipping elements in the Earth system.

To capture more pairwise interactions among multiple tipping elements and incorporate the influence of global warming, as a time-dependent external driver, such as the global mean surface temperature T varying with time t. Wunderling *et al.* [12] proposed a stylized dynamic network model to describe cascading effects among climate tipping elements. In contrast to the deterministic and autonomous framework in Eq. (10), this model is nonautonomous. Moreover, it extends beyond the interaction between two tipping elements to formulate a dynamic network framework that encompasses multiple elements under temporally varying external forcing. The model assumes a double-fold bifurcation with a linear coupling term [81,86,87]:

$$\frac{dx_i}{dt} = \left[-x_i^3 + x_i + \sqrt{\frac{4}{27}} \cdot \frac{\Delta\mathrm{GMT}(t)}{T_{\mathrm{cri},i}} + d \cdot \sum_{j \neq i} \frac{s_{ij}}{10}(x_j + 1) \right] \frac{1}{\tau_i}. \tag{11}$$

Here, the first three terms represent the intrinsic bifurcation dynamics of a single tipping element x_i, while the last term captures the interactions from other elements j. The parameter $T_{\mathrm{cri},i}$ denotes the estimated critical threshold of tipping element i, and $\Delta\mathrm{GMT}(t)$ is the increase in global mean surface temperature relative to preindustrial levels, which has been generalized to include the GMT feedback induced by the crossing of a tipping point [88]. In the coupling term, d represents the overall interaction strength, and s_{ij} quantifies the connection strength between tipping elements.

The sign of the coupling coefficient s_{ij} indicates the nature of the interaction between tipping elements: positive values denote destabilizing effects, while negative ones represent stabilizing feedbacks. As summarized in Fig. 3 and reviewed in Ref. [84], many assessed links are destabilizing, but the network is not uniformly amplifying. In particular, a weakened AMOC can enhance the stability of both the GIS and Arctic sea ice by reducing northward heat transport and promoting regional cooling. This distinction matters because the net cascade risk depends on the balance between destabilizing and compensatory links rather than on coupling alone. At the same time, the network model should be interpreted as a stylized representation instead of a fully empirically validated prediction tool. On one hand, the parameters of this model, such as $T_{\mathrm{cri},i}$, d, s_{ij}, and τ_i, rely on expert knowledge with uncertainty rather than observations or data-driven methods. Its strength lies in exploring how plausible feedback loops, interaction topologies, timescale heterogeneity, and overshoot forcing reshape the systemic risk. Its weakness is that critical thresholds $T_{\mathrm{cri},i}$, interaction weights s_{ij}, and effective coupling strengths remain only partly constrained by observations and process-based models. We therefore use this framework primarily as a conceptual and sensitivity-analysis device, while reserving stronger empirical claims for links that are independently supported by physical understanding, observations, or dedicated simulations.

Compared with the fold-bifurcation model in Eq. (4), an additional parameter τ_i is introduced to represent the characteristic timescale of tipping. Parameter values for $T_{\mathrm{cri},i}$, s_{ij}, and τ_i can be obtained from existing studies [13], while d is typically varied within the range $[0, 1]$ to account for uncertainty in real-world coupling strength. In the absence of coupling, the system approaches its bifurcation threshold as ΔGMT rises toward $T_{\mathrm{cri},i}$. When coupling is introduced, the timing of tipping events can either advance or be delayed, depending on the net effect of positive and negative links. Through large ensembles of Monte Carlo simulations propagating parameter uncertainties, the authors investigated cascading tipping among four key elements, the GIS and WAIS, the AMOC, and the Amazon rainforest. Their findings reveal that interactions tend to destabilize the network, with the polar ice sheets often acting as initiators of cascading transitions. This aligns with evidence suggesting that several cryospheric tipping elements are already approaching critical thresholds [9,89]. Nevertheless, these results should still be read as a scenario-based risk amplification under a conceptual model, not as direct observational proof that the corresponding cascade sequence has already been realized in the Earth system.

The network model in Eq. (11) was further extended to assess tipping risks under global temperature overshoot scenarios [90], in which temporary exceedance of the 1.5°C or 2°C targets, the so-called “overshoot”, substantially elevates the probability of irreversible cascading events. Using the stylized network model, the authors demonstrated that triggering one tipping element (e.g., GIS collapse) during an over-

shoot phase can "lock in" that change, subsequently destabilizing other elements (e.g., the AMOC) by reducing their tipping thresholds. This process greatly increases the likelihood of a domino-like chain of climate system failures. Crucially, their analysis shows that temperature overshoots make damaging cascades roughly three times more likely than pathways that stabilize temperatures without an overshoot. They further estimate that temporary overshoots can increase tipping risks by up to 72% compared with non-overshoot scenarios, even if the long-term equilibrium temperature remains within the Paris Agreement range. These results underscore that avoiding even temporary exceedance of critical warming limits is essential to prevent self-reinforcing and potentially irreversible climate tipping cascades.

Challenges

In this review, we have reviewed the fundamental concepts and recent advances in the study of tipping points and cascading effects from the perspective of dynamical systems and complexity science, with particular emphasis on EWS and their detection. A stylized network-based dynamic model was introduced to describe interactions and cascading behaviors among multiple climate tipping elements. Nevertheless, research in this field still faces substantial challenges, which can be broadly categorized into two aspects: *data* and *methodology*.

1. Data quantity

Thus far, most studies remain largely theoretical rather than data-driven, primarily due to the scarcity and heterogeneity of high-quality observational datasets suitable for diagnosing tipping points. A major challenge lies in the stringent data requirements of EWS methods, which typically demand long, high-resolution, and continuous time series. In practice, however, many climate records are either too short or contain missing values, resulting in discontinuities that severely hinder reliable EWS estimation. This limitation is particularly pronounced in the context of ocean observations, which are inherently sparse, spatially biased, and subject to substantial measurement uncertainties [91,92]. A key limitation arises from the uneven spatial distribution of oceanic observations across the globe, with vast data gaps in remote and deep-sea regions. For example, the Argo program [93], consisting of over 3,000 autonomous profiling floats that continuously measure temperature and salinity, represents a cornerstone of the modern ocean observing system and complements satellite-based missions. Nevertheless, significant challenges persist: observational coverage remains biased toward the Northern Hemisphere due to logistical and funding constraints, while float operations are limited in the Southern Ocean and polar regions because of technical factors such as the lack of effective ice-avoidance mechanisms. These factors collectively lead to pronounced spatial heterogeneity and substantial observational gaps [94]. Moreover, direct observations of the deep ocean are intrinsically challenging. Extreme pressure, perpetual darkness, and inaccessibility make it exceptionally difficult to obtain high-precision, high-resolution in-situ measurements in abyssal environments. Consequently, available datasets are often biased or error-prone and are nonetheless used to initialize and validate large-scale climate models. This dependence inevitably compromises the reliability of model simulations and amplifies uncertainties in future climate projections, particularly in processes such as deep and bottom water formation, which remain poorly constrained in most models [95].

In addition, all measurement systems are subject to instrumental errors, sensor drift, and calibration biases that can introduce systematic inaccuracies. Uncertainties also arise during the data processing and integration stages. For instance, gridding sparse point measurements or deriving geophysical quantities from satellite signals inevitably involves interpolation assumptions and empirical models that propagate error. Integrating heterogeneous datasets, from satellites, in situ measurements, and reanalysis products, further requires rigorous cross-calibration and homogenization to ensure consistency across spatial and temporal scales [94,96]. Moreover, uncertainties in marine ecosystem projections arise from multiple sources, including structural (model) uncertainty, initialization and internal variability, parameter uncertainty, and scenario dependence [92,97].

2. Methods of EWS and cascade

Despite their theoretical appeal, EWS methodologies face substantial practical and conceptual limitations that constrain their predictive reliability [31,56,98,99]. Dakos *et al.* [56] noted that not all regime shifts correspond to true tipping points, meaning that classical CSD-based indicators may fail to manifest even when abrupt transitions occur. A recent meta-analysis [31] reviewing over 200 studies found mixed results,

with many reporting poor or negative predictive performance, especially in ecological contexts. Notably, nearly all negative cases relied solely on CSD-based early warnings. In a large scale empirical test, Burthe *et al.* [98] analyzed long term abundance time series from 55 taxa (126 datasets) across multiple trophic levels in marine and freshwater ecosystems. Their findings revealed that true positives were rare, only 9% (16 of 170) for variance and 13% (19 of 152) for autocorrelation, while false positives occurred more frequently than false negatives (53% vs. 38% for variance; 47% vs. 40% for autocorrelation). False detections were observed across all decades and ecosystem types. Additionally, most EWS techniques are developed for single external drivers, and their fundamental assumptions can fail when multiple drivers co-vary or interact [99].

To overcome the limitations of univariate CSD indicators, particularly for detecting interactions and cascading transitions, advanced multivariate methodologies have been proposed. Techniques such as DCCA [61] extend DFA to quantify cross-correlations between non-stationary signals. Despite their theoretical advantages, these methods present the following profound practical limitations: (i). First, DCCA remains highly susceptible to spurious crossovers induced by underlying trends (e.g., linear or periodic), which can fundamentally distort scaling results [100]. (ii). Second, DCCA detects correlation strength but provides limited insight into directional causality, making it difficult to distinguish genuine coupling between tipping elements from spurious cross-correlations induced by shared external forcings [101]. Alternative causality-based approaches, such as Granger causality and convergence cross mapping (CCM), partially address this by focusing on directional inference. However, they suffer from their own shortcomings: Granger causality typically assumes linear relationships, while CCM, though capable of capturing nonlinearity, requires massive data volumes and is highly sensitive to noise [102,103]. (iii). More generally, these multivariate and network-based indicators face substantial data requirements, demanding long, high-resolution time series that are rarely available in empirical ecological or Earth system observations [104]. Consequently, their performance in real-world cascading transitions remains poorly understood compared to idealized simulated models [105], underscoring the pressing need for robust, data-efficient indicators capable of capturing nonlinear, multi-scalar interactions.

A further challenge in studying cascading tipping phenomena lies in the parameterization of stylized network models [12]. In most existing frameworks, the estimation of critical thresholds and coupling strengths relies heavily on expert judgment rather than data-driven inference, which inevitably introduces subjectivity and constrains the generalizability of these models. For example, expert assessments have suggested that tropical coral reef systems may approach a tipping point when global mean temperature exceeds approximately 1.5°C above pre-industrial levels [8]. However, widespread coral bleaching events have already been observed at warming levels of around 1.2°C [14], highlighting substantial uncertainty in expert-based estimates of critical thresholds and suggesting that some systems may respond more sensitively than previously assumed. At present, methods for empirically constraining such parameters from observed or simulated cascade events remain largely underdeveloped. Further complexity arises from the multi-timescale interactions among different climatic tipping elements, which challenge both the formulation and calibration of network-based dynamical models. This methodological gap represents a fundamental limitation: without objective, data-informed calibration, stylized network models possess limited predictive capability and reduced practical utility for assessing systemic risks or designing effective resilience and mitigation strategies.

Opportunities

Given these challenges, new methodological paradigms are urgently needed. In particular, the integration of artificial intelligence (AI) and complex network science [106] offers a promising avenue for advancing the study of tipping points and cascading transitions.

1. Artificial intelligence

Recent advances in AI have rapidly accelerated its application in ESS, leading to the emergence of an interdisciplinary AI–Earth Science research community [107–109]. Discriminative models, such as Graph Convolutional Networks and Transformers, can automatically extract complex spatiotemporal dependencies from large-scale observational and reanalysis datasets, significantly improving the accuracy and efficiency of deterministic predictions [110,111].

Crucially, to address the challenge of current data scarcity, AI frameworks are evolving

to harmonize disparate data scales. Generative AI approaches, including diffusion models and generative adversarial networks, have begun to transcend fundamental limitations of traditional data assimilation [112–115]. These models can learn the posterior distribution of meteorological states, facilitating multi-fidelity learning that fuses abundant low-fidelity data (e.g., satellite proxies) with sparse high-fidelity measurements. Such advances collectively enhance the capacity to dynamically assimilate heterogeneous observations, improving data completeness, resolution, and spatiotemporal consistency, which are key prerequisites for a robust tipping point analysis.

Beyond data processing, AI holds significant promise for advancing the detection and prediction of tipping points in complex systems. Its advantages are reflected in three main aspects. (i). First, the integration of deep learning with generative techniques significantly enhances the robustness of EWS detection frameworks. By synthesizing realistic surrogate data to augment limited historical observations [116], these approaches enable models to learn from a vast array of simulated critical regimes. This data-rich training environment allows deep learning algorithms to substantially improve both the sensitivity and specificity of tipping point detection, while maintaining strong generalizability across diverse and previously unseen systems [70]. (ii). Second, AI methods are particularly well-suited for capturing R-tipping events that are often missed by conventional EWS approaches, thereby enabling the detection of nonlinear abrupt transitions [71]. (iii). Third, advanced architectures allow for predictive modeling that extends beyond traditional EWS, enabling not only the estimation of tipping likelihoods but also the inference of critical thresholds [117,118].

While AI has emerged as a powerful tool for analyzing complex systems, many current data-driven models often function as "black boxes", offering limited insight into their internal decision-making processes [119,120]. Moreover, purely data-driven approaches may lack physical consistency, a critical limitation when applied to sparsely observed Earth system processes, such as marine tipping behavior, where training data rarely covers extreme regimes. Despite their impressive predictive capabilities, many AI-based Earth system models remain difficult to interpret mechanistically. Existing explainable AI (XAI) techniques, such as saliency maps or attention analyses, are often fundamentally correlation-based and may fail to identify the true causal drivers underlying tipping dynamics [121,122]. This issue is particularly critical for Earth system applications, where mechanistic understanding is essential for scientific credibility and policy-relevant decision-making. Furthermore, because observational records predominantly sample quasi-stable climate states, AI models may exhibit a limited extrapolation capability, when confronted with previously unseen critical transitions or cascading tipping events. Generative approaches may also inherit structural biases from climate simulations or reanalysis datasets, potentially leading to overconfident predictions in poorly observed regimes. Reliable uncertainty quantification therefore remains a major challenge for AI-driven tipping point prediction systems [123]. Future progress will likely require a tighter integration between explainable AI, causal inference frameworks, and physically grounded learning architectures to distinguish genuine EWS from spurious statistical correlations [124,125]. A promising pathway to overcome these limitations is the integration of physical laws directly into the learning architecture, an approach broadly termed *scientific machine learning* [126].

Two complementary strategies have emerged within this paradigm. (i). First, *physics-informed neural networks* (PINN) embed governing equations or conservation principles directly into the loss function as soft constraints, ensuring that predictions adhere to known physics even in data-sparse regions [127]. Nevertheless, embedding physical constraints does not fully guarantee physically faithful behavior, particularly when the governing equations are themselves uncertain or only partially known. Many PINN-based approaches also face optimization and scalability challenges in high-dimensional geophysical systems. (ii). Second, structural priors can be encoded as inductive biases within the network architecture itself. For instance, GraphCast utilizes a mesh-based graph neural network to represent long-range spatial interactions on a sphere, significantly extending the skill of medium-range weather forecasts [111]. Beyond improving predictive accuracy, progress in XAI offers a critical avenue to demystify these models, identifying the underlying drivers of tipping dynamics [128–130]. In an operational climate risk assessment, improving interpretability and trustworthiness is particularly important because black-box predictions alone may be insufficient to support robust decision-making and climate adaptation strate-

gies. Looking forward, the development of such interpretable, physics-aware AI systems represents an essential step toward building generalizable and trustworthy intelligence capable of simulating the complex, interconnected behavior of the Earth system, thereby offering critical insights into the study of its tipping points and cascading effects.

2. Complex networks method

In parallel, complex network theory has emerged as a powerful tool for analyzing the structure and dynamics of the Earth system [85,131–136]. Pioneering works have established the utility of climate networks for forecasting major climate phenomena, most notably the El Niño–Southern Oscillation (ENSO). For instance, by analyzing the cooperative behavior and link density of the climate network in the Pacific basin, Gozolchiani *et al.* [137] and Ludescher *et al.* [138] demonstrated that network-based indicators could predict the onset of El Niño events over a year in advance, which significantly outperforms conventional dynamical models. These foundational studies highlight how topological changes in the functional network, such as the localization of teleconnections, serve as robust precursors for large-scale system transitions [139].

Beyond forecasting specific events, network approaches are increasingly pivotal for identifying critical thresholds and modeling cascading transitions among tipping elements. Recent research has moved from static topology to dynamic resilience. For example, Jiang *et al.* [140] reduced high-dimensional network data to low-dimensional dynamical forms to accurately predict tipping points. Network-based metrics, including normalized degree and path length, have also proven effective in detecting global tipping behaviors [73] and capturing teleconnections, such as the atmospheric bridge linking the Amazon rainforest, the Tibetan Plateau, and the WAIS [11]. Crucially, the risk of cascading transitions, where a tipping event in one subsystem triggers a domino effect, can be validly described by models of interdependent networks and interacting tipping elements [12,141]. Looking forward, integrating these network-based mechanistic insights with AI methodologies offers a powerful, data-rich framework for not only detecting isolated tipping points but also mapping the complex pathways of systemic collapse in the Earth system.

Funding

This work was supported by the National Natural Science Foundation of China (Grant No. T2525011, 42450183, 12275020, 12135003, 42461144209, 12505036), the National Key R&D Program of China (2025YFF0517304). J.F. is supported by the Fundamental Research Funds for the Central Universities.

REFERENCES

1. Bindoff NL, Willebrand J, Artale V *et al.* Observations: Oceanic climate change and sea level. *Climate Change 2007: The Physical Science Basis. Contribution of Working Group I*, 385–428 (Cambridge University Press).
2. Gruber N, Clement D, Carter BR *et al.* The oceanic sink for anthropogenic co_2 from 1994 to 2007. *Science* 2019; **363**: 1193–1199.
3. Mora C, Tittensor DP, Adl S *et al.* How many species are there on earth and in the ocean? *PLoS biology* 2011; **9**: e1001127.
4. Organisation for Economic Co-operation and Development (OECD). The ocean economy in 2050.
5. Heinze C, Blenckner T, Martins H *et al.* The quiet crossing of ocean tipping points. *Proceedings of the National Academy of Sciences* 2021; **118**: e2008478118.
6. Carstensen J, Andersen JH, Gustafsson BG *et al.* Deoxygenation of the baltic sea during the last century. *Proceedings of the National Academy of Sciences* 2014; **111**: 5628–5633.
7. Steffen W, Richardson K, Rockström J *et al.* The emergence and evolution of earth system science. *Nature Reviews Earth & Environment* 2020; **1**: 54–63.
8. Lenton TM, Held H, Kriegler E *et al.* Tipping elements in the Earth's climate system. *Proceedings of the National Academy of Sciences* 2008; **105**: 1786–1793.
9. Lenton TM, Rockström J, Gaffney O *et al.* Climate tipping points — too risky to bet against. *Nature* 2019; **575**: 592–595.
10. Scheffer M. *Critical transitions in nature and society*, (Princeton University Press2009).
11. Liu T, Chen D, Yang L *et al.* Teleconnections among tipping elements in the earth system. *Nature Climate Change* 2023; **13**: 67–74.
12. Wunderling N, Donges JF, Kurths J *et al.* Interacting tipping elements increase risk of climate domino effects under global warming. *Earth System Dynamics* 2021; **12**: 601–619.
13. Armstrong McKay DI, Staal A, Abrams JF *et al.* Exceeding 1.5 ℃ global warming could trigger multiple climate tipping points. *Science* 2022; **377**: eabn7950.
14. Lenton TM, Milkoreit M, Willcock S *et al.* Global tipping points report 2025 2025. Available at the Global Systems Institute, University of Exeter.
15. Gladwell M. *The tipping point: How little things can make a big difference*, (Little, Brown2000).
16. Alley RB, Marotzke J, Nordhaus WD *et al.* Abrupt Climate Change. *Science* 2003; **299**: 2005–2010.
17. Alley RB, Meese D, Shuman C *et al.* Abrupt increase in greenland snow accumulation at the end of the younger dryas event. *Nature* 1993; **362**: 527–529.

18. Broecker WS. Unpleasant surprises in the greenhouse? *Nature* 1987; **328**: 123–126.
19. Masson-Delmotte V, Zhai P, Pirani A *et al. Climate Change 2021: The Physical Science Basis*, (Cambridge University Press2021). Accessed: 2024-11-20.
20. Parry IM, Ritchie PD and Cox PM. Evidence of localised amazon rainforest dieback in cmip6 models. *Earth System Dynamics* 2022; **13**: 1667–1675.
21. Flores BM, Montoya E, Sakschewski B *et al.* Critical transitions in the amazon forest system. *Nature* 2024; **626**: 555–564.
22. Ashwin P, Wieczorek S, Vitolo R *et al.* Tipping points in open systems: bifurcation, noise-induced and rate-dependent examples in the climate system. *Philosophical Transactions of the Royal Society A: Mathematical, Physical and Engineering Sciences* 2012; **370**: 1166–1184.
23. Fraedrich K. Catastrophes and resilience of a zero-dimensional climate system with ice-albedo and greenhouse feedback. *Quarterly Journal of the Royal Meteorological Society* 1979; **105**: 147–167.
24. Sutera A. On stochastic perturbation and long-term climate behaviour. *Quarterly Journal of the Royal Meteorological Society* 1981; **107**: 137–151.
25. The simulation code and plotting code for various type of tipping dynamics and early-warning signals on the Github through `https://github.com/ShengFangBNU/Tipping-points-and-Cascading-transitions`.
26. Lenton TM, Benson S, Smith T *et al.* Operationalising positive tipping points towards global sustainability. *Global Sustainability* 2022; **5**: e1.
27. Mercure JF, Lam A, Buxton JE *et al.* Evidence of a cascading positive tipping point towards electric vehicles 2026; **17**: 240.
28. Nijsse F, Sharpe S, Sahastrabuddhe R *et al.* How a positive tipping point cascade in power, transport and heating can accelerate the low-carbon transition. *Research Square* 2025; PREPRINT (Version 1).
29. Shi J, Li T and Chen L. Towards a critical transition theory under different temporal scales and noise strengths. *Physical Review E* 2016; **93**: 032137.
30. Yu Z, Liang Z, Wang J *et al.* Unified description model and typology classification of coastal tipping points. *Science China Earth Sciences* 2025; **68**: 3482–3494.
31. Dakos V, Boulton CA, Buxton JE *et al.* Tipping point detection and early warnings in climate, ecological, and human systems. *Earth System Dynamics* 2024; **15**: 1117–1135.
32. Scheffer M, Bascompte J, Brock WA *et al.* Early-warning signals for critical transitions. *Nature* 2009; **461**: 53–59.
33. Dekker MM, Von Der Heydt AS and Dijkstra HA. Cascading transitions in the climate system. *Earth System Dynamics* 2018; **9**: 1243–1260.
34. Boers N. Observation-based early-warning signals for a collapse of the atlantic meridional overturning circulation. *Nature Climate Change* 2021; **11**: 680–688.
35. Held H and Kleinen T. Detection of climate system bifurcations by degenerate fingerprinting. *Geophysical Research Letters* 2004; **31**: L23207.
36. Peng CK, Buldyrev SV, Havlin S *et al.* Mosaic organization of dna nucleotides. *Physical Review E* 1994; **49**: 1685.
37. Livina VN and Lenton TM. A modified method for detecting incipient bifurcations in a dynamical system. *Geophysical Research Letters* 2007; **34**: L03712.
38. Boers N, Liu T, Bathiany S *et al.* Destabilization of Earth system tipping elements. *Nature Geoscience* 2025; **18**: 949–960.
39. Boulton CA, Lenton TM and Boers N. Pronounced loss of amazon rainforest resilience since the early 2000s. *Nature Climate Change* 2022; **12**: 271–278.
40. Dakos V, van Nes EH, Donangelo R *et al.* Spatial correlation as leading indicator of catastrophic shifts. *Theoretical Ecology* 2010; **3**: 163–174.
41. Guttal V and Jayaprakash C. Spatial variance and spatial skewness: leading indicators of regime shifts in spatial ecological systems. *Theoretical Ecology* 2009; **2**: 3–12.
42. Dai L, Korolev KS and Gore J. Slower recovery in space before collapse of connected populations. *Nature* 2013; **496**: 355–358.
43. Kéfi S, Guttal V, Brock WA *et al.* Early warning signals of ecological transitions: methods for spatial patterns. *PLoS ONE* 2014; **9**: e92097.
44. Smith T, Morr A, Bookhagen B *et al.* Predicting instabilities in transient landforms and interconnected ecosystems. *Nature Communications* 2026; **17**: 1316.
45. Guttal V and Jayaprakash C. Changing skewness: An early warning signal of regime shifts in ecosystems. *Ecology Letters* 2008; **11**: 450–460.
46. Wang R, Dearing JA, Langdon PG *et al.* Flickering gives early warning signals of a critical transition to a eutrophic lake state. *Nature* 2012; **492**: 419–422.
47. Mayer AL, Pawlowski CW and Cabezas H. Fisher information and dynamic regime changes in ecological systems. *Ecological Modelling* 2006; **195**: 72–82.
48. Ahmad N, Derrible S, Eason T *et al.* Using fisher information to track stability in multivariate systems. *Royal Society Open Science* 2016; **3**: 160582.
49. Eason T, Garmestani AS and Cabezas H. Managing for resilience: early detection of regime shifts in complex systems. *Clean Technologies and Environmental Policy* 2014; **16**: 773–783.
50. Karunanithi AT, Cabezas H, Frieden BR *et al.* Detection and assessment of ecosystem regime shifts from fisher information. *Ecology and Society* 2008; **13**: 22.
51. Sundstrom SM, Eason T, Nelson RJ *et al.* Detecting spatial regimes in ecosystems. *Ecology Letters* 2017; **20**: 19–32.
52. Dakos V, Scheffer M, Van Nes EH *et al.* Slowing down as an early warning signal for abrupt climate change. *Proceedings of the National Academy of Sciences* 2008; **105**: 14308–14312.
53. Kleinen T, Held H and Petschel-Held G. The potential role of spectral properties in detecting thresholds in the earth system: application to the thermohaline circulation. *Ocean Dynamics* 2003; **53**: 53–63.
54. Chen S, O'Dea EB, Drake JM *et al.* Eigenvalues of the covariance matrix as early warning signals for critical transitions in ecological systems. *Scientific Reports* 2019; **9**: 2572.
55. Grziwotz F, Chang CW, Dakos V *et al.* Anticipating the occurrence and type of critical transitions. *Science Advances* 2023; **9**: eabq4558.
56. Dakos V, Carpenter SR, van Nes EH *et al.* Resilience indicators: prospects and limitations for early warnings of regime shifts. *Philosophical Transactions of the Royal Society B: Biological Sciences* 2015; **370**: 20130263.

57. Prettyman J, Kuna T and Livina V. Generalized early warning signals in multivariate and gridded data with an application to tropical cyclones. *Chaos: An Interdisciplinary Journal of Nonlinear Science* 2019; **29**: 073105.
58. Lade SJ and Gross T. Early warning signals for critical transitions: A generalized modeling approach. *PLoS Computational Biology* 2012; **8**: e1002360.
59. Dakos V, Carpenter SR, Brock WA *et al.* Methods for detecting early warnings of critical transitions in time series illustrated using simulated ecological data. *PloS one* 2012; **7**: e41010.
60. Laitinen V, Dakos V and Lahti L. Probabilistic early warning signals. *Ecology and Evolution* 2021; **11**: 14101–14114.
61. Podobnik B and Stanley HE. Detrended cross-correlation analysis: A new method for analyzing two nonstationary time series. *Physical review letters* 2008; **100**: 084102.
62. Livina VN, Kwasniok F and Lenton TM. Potential analysis reveals changing number of climate states during the last 60 kyr. *Climate of the Past* 2010; **6**: 77–82.
63. Seekell DA, Carpenter SR and Pace ML. Conditional heteroscedasticity as a leading indicator of ecological regime shifts. *American Naturalist* 2011; **178**: 442–451.
64. Xu L, Patterson D, Staver AC *et al.* Unifying deterministic and stochastic ecological dynamics via a landscape-flux approach. *Proceedings of the National Academy of Sciences of the United States of America* 2021; **118**: e2103779118.
65. Xu L, Patterson DD, Levin SA *et al.* Global stability and tipping point prediction in a coral–algae model using landscape–flux theory. *Earth System Dynamics* 2025; **16**: 1503–1522.
66. Kefi S, Rietkerk M, Alados C *et al.* Spatial vegetation patterns and imminent desertification in mediterranean arid ecosystems. *Nature* 2007; **449**: 213–217.
67. Pavithran I and Sujith RI. Effect of rate of change of parameter on early warning signals for critical transitions. *Chaos: An Interdisciplinary Journal of Nonlinear Science* 2021; **31**: 013116.
68. Clements CF and Ozgul A. Including trait-based early warning signals helps predict population collapse. *Nature Communications* 2016; **7**: 10984.
69. Dakos V, Carpenter SR, Brock WA *et al.* Methods for Detecting Early Warnings of Critical Transitions in Time Series Illustrated Using Simulated Ecological Data. *PLOS ONE* 2012; **7**: e41010.
70. Bury TM, Sujith RI, Pavithran I *et al.* Deep learning for early warning signals of tipping points. *Proceedings of the National Academy of Sciences* 2021; **118**: e2106140118.
71. Huang Y, Bathiany S, Ashwin P *et al.* Deep learning for predicting rate-induced tipping. *Nature Machine Intelligence* 2024; **6**: 1556–1565.
72. Chen L, Liu R, Liu ZP *et al.* Detecting early-warning signals for sudden deterioration of complex diseases by dynamical network biomarkers. *Scientific Reports* 2012; **2**: 342.
73. Moinat L, Kasparian J and Brunetti M. Tipping detection using climate networks. *Chaos: An Interdisciplinary Journal of Nonlinear Science* 2024; **34**: 123161.
74. Hastings A, Petrovskii S, Lucarini V *et al.* Tipping points in complex ecological systems. *arXiv* 2026; .
75. Donovan GM and Brand C. Spatial early warning signals for tipping points using dynamic mode decomposition. *Physica A: Statistical Mechanics and its Applications* 2022; **596**: 127152.
76. Rocha JC, Peterson G, Bodin Ö *et al.* Cascading regime shifts within and across scales. *Science* 2018; **362**: 1379–1383.
77. Klose AK, Wunderling N, Winkelmann R *et al.* What do we mean, 'tipping cascade'? *Environmental Research Letters* 2021; **16**: 125011.
78. Wunderling N, Gelbrecht M, Winkelmann R *et al.* Basin stability and limit cycles in a conceptual model for climate tipping cascades. *New Journal of Physics* 2020; **22**: 123031.
79. Tigchelaar M, Von Der Heydt A and Dijkstra H. A new mechanism for the two-step δ^{18}o signal at the eocene-oligocene boundary. *Climate of the Past* 2011; **7**: 235–247.
80. van Nes EH and Scheffer M. Implications of spatial heterogeneity for catastrophic regime shifts in ecosystems. *Ecology* 2005; **86**: 1797–1807.
81. Brummitt CD, Barnett G and D'Souza RM. Coupled catastrophes: sudden shifts cascade and hop among interdependent systems. *Journal of The Royal Society Interface* 2015; **12**: 20150712.
82. Basak A, Dana SK and Bairagi N. Cascade tipping, cascade hopping and indirect tipping in ecological systems. *Physical Review E* 2025; **112**: 044225.
83. Ashwin P, Bastiaansen R, von der Heydt AS *et al.* Early warning skill, extrapolation and tipping for accelerating cascades. *Proceedings of the Royal Society A: Mathematical, Physical and Engineering Sciences* 2025; **481**: 20250405.
84. Wunderling N, von der Heydt AS, Aksenov Y *et al.* Climate tipping point interactions and cascades: A review. *Earth System Dynamics* 2024; **15**: 41–74.
85. Boers N, Goswami B, Rheinwalt A *et al.* Complex networks reveal global pattern of extreme-rainfall teleconnections. *Nature* 2019; **566**: 373–377.
86. Klose AK, Karle V, Winkelmann R *et al.* Emergence of cascading dynamics in interacting tipping elements of ecology and climate. *Royal Society open science* 2020; **7**: 200599.
87. Krönke J, Wunderling N, Winkelmann R *et al.* Dynamics of tipping cascades on complex networks. *Physical Review E* 2020; **101**: 042311.
88. Bdolach T, Kurths J and Yanchuk S. Tipping in an adaptive climate network model. *Chaos: An Interdisciplinary Journal of Nonlinear Science* 2025; **35**: 053157.
89. Pörtner HO, Roberts DC, Masson-Delmotte V *et al.* The ocean and cryosphere in a changing climate. *IPCC special report on the ocean and cryosphere in a changing climate* 2019; **1155**: 10–1017.
90. Wunderling N, Winkelmann R, Rockström J *et al.* Global warming overshoots increase risks of climate tipping cascades in a network model. *Nature Climate Change* 2023; **13**: 75–82.
91. Elipot S, Drushka K, Subramanian A *et al.* Overcoming the challenges of ocean data uncertainty. *Eos (Washington, DC)* 2022; **103**.
92. Kennedy JJ. A review of uncertainty in in situ measurements and data sets of sea surface temperature. *Reviews of Geophysics* 2014; **52**: 1–32.

93. Jayne SR, Roemmich D, Zilberman N *et al.* The argo program: Present and future. *Oceanography* 2017; **30**: 18–28.
94. Abraham JP, Baringer M, Bindoff NL *et al.* A review of global ocean temperature observations: Implications for ocean heat content estimates and climate change. *Reviews of Geophysics* 2013; **51**: 450–483.
95. Heuzé C. Antarctic bottom water and north atlantic deep water in CMIP6 models. *Ocean Science Discussions* 2020; **2020**: 1–38.
96. Smith T, Zotta RM, Boulton CA *et al.* Reliability of resilience estimation based on multi-instrument time series. *Earth System Dynamics Discussions* 2022; **2022**: 1–14.
97. Payne MR, Barange M, Cheung WW *et al.* Uncertainties in projecting climate-change impacts in marine ecosystems. *ICES Journal of Marine Science* 2016; **73**: 1272–1282.
98. Burthe SJ, Henrys PA, Mackay EB *et al.* Do early warning indicators consistently predict nonlinear change in long-term ecological data? *Journal of Applied Ecology* 2016; **53**: 666–676.
99. Dai L, Korolev KS and Gore J. Relation between stability and resilience determines the performance of early warning signals under different environmental drivers. *Proceedings of the National Academy of Sciences* 2015; **112**: 10056–10061.
100. Zhao X, Shang P, Zhao C *et al.* Minimizing the trend effect on detrended cross-correlation analysis with empirical mode decomposition. *Chaos, Solitons & Fractals* 2011; **44**: 185–190.
101. Weinans E, Quax R, van de Leemput IA *et al.* Evaluating early-warning indicators for tipping points in nonlinear multivariate networks. *Journal of the Royal Society Interface* 2021; **18**: 20210338.
102. Bian S *et al.* Utilizing causal network markers to identify tipping points ahead of critical transition. *Advanced Science* 2025; **12**: e202415732.
103. Runge J *et al.* Detecting causal associations in large nonlinear time series datasets. *Science Advances* 2023; **9**: eadd6896.
104. West R *et al.* Comparing early warning signal performance in spatially-structured systems. *PLOS Computational Biology* 2024; **20**: e1012509.
105. George SV, Kachhara S and Ambika G. Early warning signals for critical transitions in complex systems: A topical review. *arXiv preprint arXiv:2107.01210* 2023; .
106. Pósfai M and Barabási AL. *Network science*, (Cambridge University Press Cambridge, UK:2016).
107. Irrgang C, Boers N, Sonnewald M *et al.* Towards neural earth system modelling by integrating artificial intelligence in earth system science. *Nature Machine Intelligence* 2021; **3**: 667–674.
108. Chen M, Qian Z, Boers N *et al.* Collaboration between artificial intelligence and earth science communities for mutual benefit. *Nature Geoscience* 2024; **17**: 949–952.
109. Bracco A, Brajard J, Dijkstra HA *et al.* Machine learning for the physics of climate. *Nature Reviews Physics* 2025; **7**: 6–20.
110. Kurth T, Subramanian S, Harrington P *et al.* Fourcastnet: Accelerating global high-resolution weather forecasting using adaptive fourier neural operators. *Proceedings of the Platform for Advanced Scientific Computing Conference* (2023) 1–11.
111. Lam R, Sanchez-Gonzalez A, Willson M *et al.* Learning skillful medium-range global weather forecasting. *Science* 2023; **382**: 1416–1421.
112. Geer AJ. Learning earth system models from observations: machine learning or data assimilation? *Philosophical Transactions of the Royal Society A* 2021; **379**: 20200089.
113. Brajard J, Carrassi A, Bocquet M *et al.* Combining data assimilation and machine learning to emulate a dynamical model from sparse and noisy observations: A case study with the lorenz 96 model. *Journal of computational science* 2020; **44**: 101171.
114. Sonnewald M, Lguensat R, Jones DC *et al.* Bridging observations, theory and numerical simulation of the ocean using machine learning. *Environmental Research Letters* 2021; **16**: 073008.
115. Martin SA, Manucharyan GE and Klein P. Generative data assimilation for surface ocean state estimation from multimodal satellite observations. *Journal of Advances in Modeling Earth Systems* 2025; **17**: e2025MS005063.
116. Ma Z, Zeng C, Zhang YC *et al.* Predicting critical transitions with machine learning trained on surrogates of historical data. *Communications Physics* 2025; **8**: 258.
117. Liu Z, Zhang X, Ru X *et al.* Early predictor for the onset of critical transitions in networked dynamical systems. *Physical Review X* 2024; **14**: 031009.
118. Zhuge C, Li J and Chen W. Deep learning for predicting the occurrence of tipping points. *Royal Society Open Science* 2025; **12**: 242240.
119. Montavon G, Binder A, Lapuschkin S *et al.* Layer-wise relevance propagation: an overview. *Explainable AI: Interpreting, Explaining and Visualizing Deep Learning* 2019; 193–209.
120. Reichstein M, Camps-Valls G, Stevens B *et al.* Deep learning and process understanding for data-driven earth system science. *Nature* 2019; **566**: 195–204.
121. Rudin C. Stop explaining black box machine learning models for high stakes decisions and use interpretable models instead. *Nature Machine Intelligence* 2019; **1**: 206–215.
122. McGovern A, Lagerquist R, Gagne II DJ *et al.* Making the black box more transparent: Understanding the physical implications of machine learning. *Bulletin of the American Meteorological Society* 2019; **100**: 2175–2199.
123. Begoli E, Bhattacharya T and Kusnezov D. The need for uncertainty quantification in machine-assisted decision making. *Nature Machine Intelligence* 2019; **1**: 20–23.
124. Pearl J and Mackenzie D. *The Book of Why: The New Science of Cause and Effect*, (Basic Books2019).
125. Peters J, Janzing D and Schölkopf B. *Elements of Causal Inference: Foundations and Learning Algorithms*, (MIT Press2017).
126. Karniadakis GE, Kevrekidis IG, Lu L *et al.* Physics-informed machine learning. *Nature Reviews Physics* 2021; **3**: 422–440.
127. Raissi M, Perdikaris P and Karniadakis GE. Physics-informed neural networks: A deep learning framework for solving forward and inverse problems involving nonlinear partial differential equations. *Journal of Computational Physics* 2019; **378**: 686–707.

128. Samek W, Montavon G, Lapuschkin S *et al.* Explainable artificial intelligence: Understanding, visualizing and interpreting deep learning models. *arXiv preprint arXiv:1708.08296* 2017; .
129. Barnes EA, Hurrell JW, Ebert-Uphoff I *et al.* Viewing forced climate patterns through an ai lens. *Geophysical Research Letters* 2019; **46**: 13389–13398.
130. Toms BA, Barnes EA and Ebert-Uphoff I. Physically interpretable neural networks for the geosciences: Applications to earth system variability. *Journal of Advances in Modeling Earth Systems* 2020; **12**: e2020MS002181.
131. Fan J, Meng J, Ludescher J *et al.* Statistical physics approaches to the complex earth system. *Physics Reports* 2021; **896**: 1–84.
132. Donges JF, Zou Y, Marwan N *et al.* Complex networks in climate dynamics. *The European Physical Journal Special Topics* 2009; **174**: 157–179.
133. Ludescher J, Gozolchiani A, Bogunov MI *et al.* Improved el niño forecasting by cooperativity detection. *Proceedings of the National Academy of Sciences* 2013; **110**: 11742–11745.
134. Tsonis AA and Roebber PJ. The architecture of the climate network. *Physica A: Statistical Mechanics and its Applications* 2004; **333**: 497–504.
135. Lu Z, Dong W, Lu B *et al.* Early warning of the indian ocean dipole using climate network analysis. *Proceedings of the National Academy of Sciences* 2022; **119**: e2109089119.
136. Feng QY and Dijkstra HA. Climate network stability measures of el niño variability. *Chaos: An Interdisciplinary Journal of Nonlinear Science* 2017; **27**: 035801.
137. Gozolchiani A, Havlin S and Yamasaki K. Emergence of el niño as an autonomous component in the climate network. *Physical Review Letters* 2011; **107**: 148501.
138. Ludescher J, Gozolchiani A, Bogachev MI *et al.* Very early warning of next el niño. *Proceedings of the National Academy of Sciences* 2014; **111**: 2064–2066.
139. Meng J, Fan J, Ashkenazy Y *et al.* Percolation framework to describe el niño conditions. *Chaos: An Interdisciplinary Journal of Nonlinear Science* 2018; **28**: 043125.
140. Jiang J, Huang ZG, Seager TP *et al.* Predicting tipping points in mutualistic networks through dimension reduction. *Proceedings of the National Academy of Sciences* 2018; **115**: E639–E647.
141. Buldyrev SV, Parshani R, Paul G *et al.* Catastrophic cascade of failures in interdependent networks. *Nature* 2010; **464**: 1025–1028.
